# Seroepidemiological study of *Toxoplasma gondii* in equines in Northern Egypt

**DOI:** 10.3389/fvets.2025.1561145

**Published:** 2025-05-19

**Authors:** Ehab Kotb Elmahallawy, Marwa F. Hassan, David Cano-Terriza, Nada Oudah Albalawi, Tomás Fajardo, Asmaa Aboelabbas Gouda, Ayman Atiba, Ahmed Hendawy, Isabelle Villena, Ashraf Mohamed Barakat, Hind Alzaylaee, Sonia Almería, Ignacio García-Bocanegra

**Affiliations:** ^1^Departamento de Sanidad Animal, Grupo de Investigación en Sanidad Animal y Zoonosis (GISAZ), Universidad de Córdoba, Córdoba, Spain; ^2^Department of Zoonoses, Faculty of Veterinary Medicine, Sohag University, Sohag, Egypt; ^3^Department of Biochemistry, Toxicology and Feed Deficiency, Animal Health Research Institute (AHRI), Agriculture Research Center (ARC), Dokki, Giza, Egypt; ^4^CIBERINFEC, ISCIII CIBER de Enfermedades Infecciosas, Instituto de Salud Carlos III, Madrid, Spain; ^5^Department of Biology, Faculty of Science, Taibah University, Al-Madinah Province, Saudi Arabia; ^6^Department of Parasitology, Faculty of Veterinary Medicine, Zagazig University, Zagazig, Egypt; ^7^Department of Surgery, Anesthesiology and Radiology, Faculty of Veterinary Medicine, Kafrelsheikh University, Kafr El Sheikh, Egypt; ^8^Laboratory of Parasitology, National Reference Centre for Toxoplasmosis, Reims Hospital, University of Reims Champagne-Ardenne, UR 7510, Reims, France; ^9^Department of Zoonotic Diseases, National Research Centre, Giza, Egypt; ^10^Department of Biology, College of Science, Princess Nourah bint Abdulrahman University, Riyadh, Saudi Arabia; ^11^Virology and Parasitology Branch, Department of Health and Human Services, Division of Food and Environmental Safety, Office of Applied Microbiology and Technology (OAMT), Office of Laboratory Operations and Applied Sciences (OLOAS), Food and Drug Administration, Laurel, MD, United States

**Keywords:** *Toxoplasma gondii*, Modified Agglutination Test (MAT), horse, donkeys, Egypt

## Abstract

**Introduction:**

Toxoplasmosis, caused by the intracellular protozoan *Toxoplasma gondii* (*T. gondii*), continues to be a widespread parasitic zoonotic disease globally. The seroepidemiology of *T. gondii* infection in Egyptian equids, particularly donkeys, remains insufficiently explored. The present study was designed to assess the seroprevalence of *T. gondii* in equines from Northern Egypt.

**Methods:**

A total of 360 serum samples from two equine species (157 horses and 203 donkeys) were obtained during 2023. The Modified Agglutination Test (MAT, cut-off of 1:25) was used to screen for the anti-*T. gondii* antibodies. The study also analyzed potential risk factors that could contribute to the exposure of the animals to the parasite, including species, breed, sex, age, and the specific location of each animal.

**Results:**

The overall seroprevalence of *T. gondii* among examined equines was 41.11% (95% Confidence Interval [CI]: 36.03–46.19). The relationships between seropositivity and explanatory variables were analyzed using a Generalized Estimating Equation (GEE) approach. The seroprevalence of *T. gondii* was significantly higher in donkeys (51.23%) than in horses (28.03%; *p* < 0.001; odds ratio [OR] = 2.99; 95% CI: 2.35–3.81).

**Conclusions:**

Collectively, our findings revealed a high *T. gondii* exposure among equine species in Northern Egypt, with a notably higher seroprevalence in donkeys compared to horses. This study represents one of the most extensive serosurveys of *T. gondii* in equids conducted in Egypt, featuring the largest sample size of donkeys examined to date. It also examined previously unexplored risk factors related to parasite exposure in equids. The present findings highlight the critical importance of performing periodical surveillance, monitoring, and management of the parasite among equids, which might have a major impact on animal and public health.

## 1 Introduction

*Toxoplasma gondii*, the causative agent of toxoplasmosis, remains one of the most common intracellular protozoa in the world. Sexual reproduction of these protozoa occurs in felids, the definitive hosts, which release oocysts in their feces. Intermediate hosts, which encompass nearly all warm-blooded species, can also become infected (1, 2). Humans and animals most commonly contract *T. gondii* through several primary routes: consuming undercooked or raw meat containing tissue cysts of the parasite, ingesting food or water contaminated with sporulated *T. gondii* oocysts, and via blood transfusions or transplacental transmission involving tachyzoites. In relation to its clinical impact, *T. gondii* infections are typically sub-clinical in immunocompetent persons; nevertheless, in immunocompromised individuals, this opportunistic protozoon may induce fatal conditions and even death as well as cause abortion, congenital malformations, and stillbirth (1).

Horses and donkeys are essential for agricultural work, transportation, and economic support, especially in rural areas, and they hold cultural and historical significance, contributing to traditional practices and local economies. However, equid production faces major challenges from various pathogens, including parasites. Among others, *T. gondii* infection in equines occurs primarily through food or water contaminated with sporulated oocysts, nonetheless, tachyzoites may also be transferred from the mare to the fetus through the placenta (3, 4). Despite *T. gondii* infection in horses being usually subclinical, atypical clinical signs of toxoplasmosis such as ataxia, fever, encephalomyelitis, and retinal degeneration have been reported (5). Abortion, fever, stillbirth, and degeneration in retina were also reported in pregnant mares infected with *T. gondii* (4, 6). Additionally, some previous works reported the potential association between clinical equine protozoal myeloencephalitis in horses and *T. gondii* seropositivity (7, 8). In the USA, fatal toxoplasmosis was observed in a horse (9). Collectively, *T. gondii* infection in equines may have a substantial impact, posing animal health risks and contributing to significant economic and reproductive losses (10). Taking this into account, consumption of equine meat is still prevalent in several countries, particularly those of the European Union (EU) (11). Previous studies have established the epidemiological link between the consumption of horse meat and clinical toxoplasmosis in humans (12). Additionally, the rising popularity of raw donkey milk has raised concerns, suggesting that consuming such milk from seropositive donkeys may increase the risk of human toxoplasmosis (13). In Egypt, horses and donkeys play a crucial role in agriculture, transportation, and economic sustainability, particularly in rural areas. Beyond their practical use, these animals also hold cultural and historical significance, contributing to traditional practices and local economies. Furthermore, raw meat of these animals is used as feed for carnivorous zoo animals, which may be represent a potential source of a source of infection if the meat is contaminated (14). In this context, viable T. gondii was detected in tissues of 25 donkeys slaughtered at the Giza Zoo abattoir (15). Clearly, infection by *T. gondii* might have an important impact on public health (10).

Monitoring exposure to the parasite is pivotal for implementation of effective control measures. Serological tests are the primary methods for diagnosing *T. gondii* in farm animals, including equids (14–16). These techniques are helpful tools for conducting screening surveys since they allow us to identify *Toxoplasma*-positive animals and farms as well as to analyze the associated risk factors linked to parasite exposure (1).

Previous literature showed that numerous serological investigations have been assessing *T. gondii* exposure in equids worldwide (7, 17). In Egypt, seroprevalence rates of *T. gondii* in horses and donkeys have been reported to vary widely, ranging from 12.0% to 68.4% (Table 1) (15, 18–25). Most previous studies involved only a limited number of animals and were performed over short periods of time. In addition, existing data on the seroprevalence of *T. gondii* in equines from Northern Egypt, particularly donkeys, remain insufficient, leaving a gap in comprehensive data. Consequently, the current study aimed to evaluate the seroprevalence of *T. gondii* and identify potential risk factors associated with exposure to this zoonotic protozoan in equids from Northern Egypt.

**Table 1:** Seroprevalence of *Toxoplasma gondii* reported in equines in Northern Egypt.

**Species**	**Source of sera**	**Governorate**	**Detection method**	**Seroprevalence % (no. pos./total)**	**Cut off value**	**Reference**
Donkeys	Rural areas	Menoufiya	ELISA	65.3 (79/121)	NS	(18)
	Individual owners	Menoufiya	LAT, ELISA	30.2(13/43), 25.6 (11/43)	1:32; 1:100	(19)
	Zoo abattoir	Giza	^*^MAT (1:25)	^a^44.5 (89/200), ^b^52.0 (104/200), ^c^36.0 (72/200), ^d^39.0 (78/200)	NS	(20)
	Individual owners	Giza	LAT, ELISA	27.6 (16/58), 37.9 (22/58)	1:32; 1:100	(19)
	Individual owners	Cairo	ELISA	45.0 (45/100)	1:200	(21)
	NS	Dakahlia	LAT, IHA, ELISA	44.3 (35/79), 67.1 (53/79), 68.4 (54/79)	NS	(15)
	Individual owners	Matrouh	LAT, ELISA	22.2 (10/45), 20.0 (9/45)	1:32; 1:100	(19)
Horses	Farms	NS	^**^ELISA-LA, ELISA-LAunb, ELISA-Lab, IFAT, MAT (1:25)	^a^38.1 (160/420), ^b^31.7 (133/420), ^c^51.7 (217/420), 40.5 (170/420), 48.1 (202/420)	NS	(22)
	Individual owners	Cairo	ELISA	25.0 (25/100)	NS	(23)
	Main farm	Cairo	LAT, MAT(1:25), ELISA	52.1 (125/240), 50.8 (122/240), 39.2 (94/240)	NS	(24)
	NS	Dakahlia	LAT, IHA, ELISA	50.0 (27/54), 72.2 (39/54), 72.2 (39/54)	NS	(15)
	Farms	Giza	ELISA	20.9 (23/110)	NS	(25)
	Farms	Qalyubia	ELISA	12.0 (12/100)	NS	(25)
	Farms	Gharbia	ELISA	14.0 (14/100)	NS	(25)
	Farms	Kafr El Sheikh	ELISA	17.3 (19/110)	NS	(25)

ELISA, enzyme-linked immunosorbent assay; LAT, latex agglutination test; MAT, modified agglutination test; IHA, indirect hemagglutination; IFAT, indirect fluorescent antibody test; NS, Not stated.

^*^MAT was carried out using ^a^RH strain, ^b^local equine strain, ^c^local camel strain and ^d^local sheep strain.

^**^ELISA was carried out using ^a^crude antigen (LA) prepared from local horse strain, and its purified immunogenetic fractions; ^b^bound (LAb) and ^c^unbound (LAunb) fractions.

## 2 Materials and methods

### 2.1 Study area

**Figure 1:**
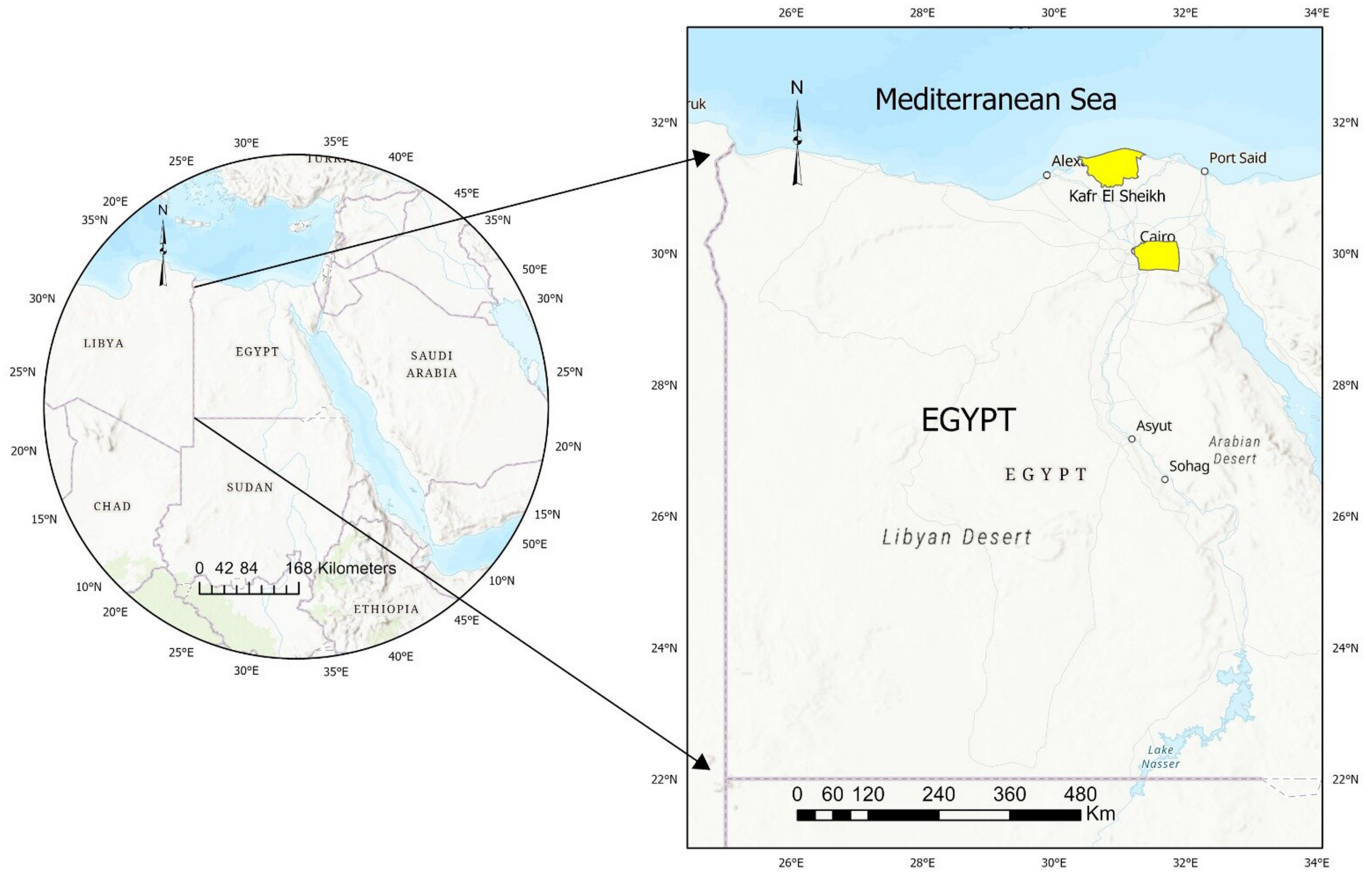
Map of Egypt showing the studied area and sampling sites.

In 2019, Egypt's equid population was reported to be 958.190, with donkeys making up 90.9% of this number, approximately 871.447 donkeys (26). The present study was conducted in Northern Egypt, primarily focusing on two regions: Cairo and Kafr El Sheikh, which are geographically at 30° 02' 30” N, 031° 14' 07” E and 31° 06' 42” N, 30° 56' 45” E respectively (Figure 1). Both regions experience mild to hot weather throughout most of the year, with average summer temperatures ranging from 31 to 34°C (88.5–92°F) and winter temperatures between 13.5 and 18°C (56.3–65°F). Northern Egypt receives an average annual rainfall of only 100–200 mm (4–8 inches), primarily occurring during the winter months.

### 2.2 Animals and samples

Between January and December 2023, blood samples were collected from 360 equines, across Northern Egypt (Figure 1). Samples were collected randomly from donkeys and horses owned by small stakeholders relying on them for transportation and agriculture, taking advantage of opportunities presented by local veterinary campaigns. Considering the number of equids in the study area (*n* > 10.000), an estimated prevalence of 36% (based on an estimated national seroprevalence threshold of 35.79% [Table 1]), an accepted error of 5% and a 95% confidence interval (95%CI) resulted in 354 animals to be sampled. A total of 360 equids, including 157 horses, 203 donkeys, were finally included in the study. A total of 10 ml blood samples were collected from each animal by puncturing the jugular vein. Sera were obtained by centrifugation at 3,000 rpm for 10 min and were kept at −20°C until serological assessment. The information on animals was thoroughly documented, whenever possible. Information collected included species, breed, sex, age, and the specific location of each animal. Age of examined animals were classified into four categories: foal animals (< 1 year), young (1–4 years), adult (4–15 years) and geriatric (>15 years) as outlined in prior studies (27, 28). The ages of the examined equines were based on the animal's dentition (29, 30).

### 2.3 Serological examination

The presence of *T. gondii* antibodies in the serum of equids was evaluated by the modified agglutination test (MAT). This method has been previously validated in equine and performed according to the protocols established by Dubey and Desmonts (31). Briefly, the test was utilized to detect *T. gondii* antibodies, employing whole-killed tachyzoites as antigens which is kindly provided by Laboratory of Parasitology, University of Reims Champagne-Ardenne, France. The protocol (31) incorporated 2-mercaptoethanol directly into the antigen rather than treating the serum. Equine serum samples were first diluted at a 1:20 ratio in phosphate-buffered saline (PBS, pH 7.2), and 0.05 mL of the diluted sample was added to U-bottom microtiter plate wells. Serial two- to fourfold dilutions were then performed using PBS. The antigen stock solution was diluted (1:10) in a freshly prepared alkaline buffer containing 2-mercaptoethanol, which was either made fresh or stored for no longer than 2 weeks. The antigen solution (0.05 mL) was then added to each well, and the plates were sealed with cellophane before being incubated overnight at 37°C in a humidified environment. The results of the assay were assessed using a microtiter plate under appropriate lighting conditions. A diffuse mat across the wellthe well indicated a positive reaction, whereas a compact button at the center signified a negative result. To ensure assay accuracy and reproducibility, each run included validated positive and negative serum controls. A titer of 1:25, was applied as a cut-off for *T. gondii* seropositivity as previously considered for these animal species (32). Additionally, serum that initially tested positive at ≥1:25 dilution was thereafter retested at 1:25 and 1:50.

### 2.4 Statistical analysis

To establish the seroprevalence of *T. gondii*, we computed the percentage of seropositive samples relative to the total number of examined equids, with a 95% confidence interval (95% CI). Relation between explanatory variables (species, breeds, sex, age, and region) and serological results was estimated using a Pearson's chi-square or Fisher exact tests to allocate the relevance of these variables in the risk of exposure of animals to *T. gondii* in a bivariate analysis. Variables with a *p* < 0.10 were selected for multivariate analysis, considering collinearity through Cramer's V coefficients. Generalized Estimating Equation (GEE) models were utilized to assess the influence of explanatory variables identified in the bivariate analysis (33), with municipality as a random effect. Statistical significance was set at *p* < 0.05, and analysis was conducted using SPSS 25.0 software.

## 3 Results

**Figure 2:**
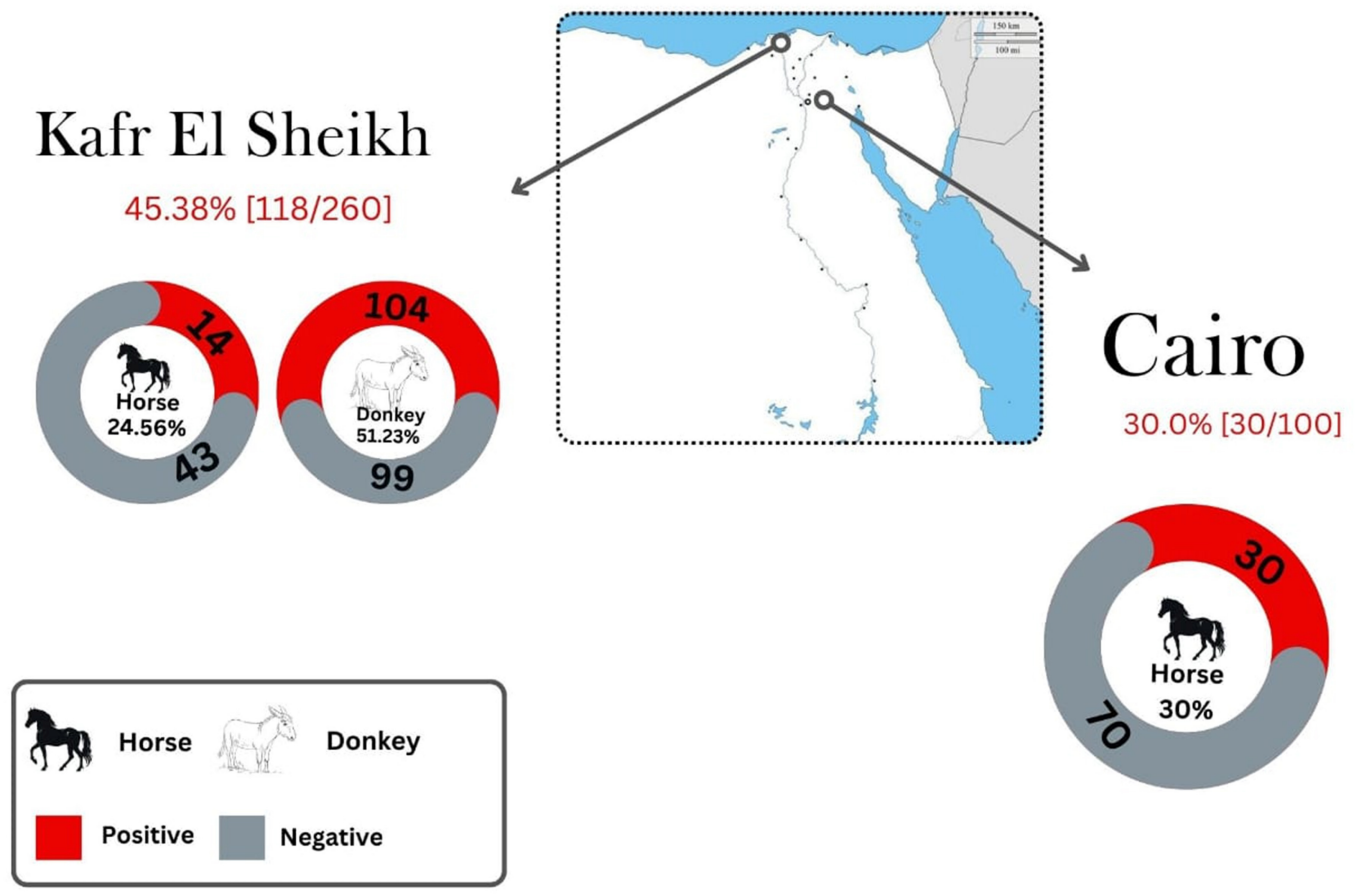
Seroprevalence of *Toxoplasma gondii* in horses and donkeys in Northern Egypt by location.

In this work, anti-*T. gondii* antibodies were detected in 148 of 360 equines (41.11%; 95% CI: 36.03–46.19). Seropositivity by species was 51.23% (104/203) in donkeys and 28.03% (44/157) in horses (Figure 2, Table 2). Among the positive samples, *T. gondii* titres in donkeys were 1:25 in 43.3% (45/104) and 1:50 in 56.3% (59/104). In horses, 54.5% (24/44) had titers of 1:25, while 45.4% (20/44) of positive animals showed titers of 1:50. Table 2 presents the distribution of *T. gondii* seropositivity, as determined by the bivariate analysis and the epidemiological questionnaire results. Two out of five explanatory variables associated (*p* < 0.10) with *T. gondii* seropositivity in equines were selected after data exploration and bivariate analyses and included in the multivariable analysis. The final GEE multivariable analysis identified species as risk factorpotentially linked with the exposure to *T. gondii* (Table 3). In this respect, significantly higher seropositivity was found in donkeys (*p* < 0.001, OR = 2.99; 95% CI: 2.35–3.81) compared to horses.

**Table 2:** Univariable analysis of risk factors associated with *T. gondii* exposure in equines.

**Variable**	**Category**	No. positives/overall^*^	**Seroprevalence (%)**	***P-*value**
Species	Donkey	104/203	51.23	< 0.001
	Horse	44/157	28.03	
Breeds	Pure breed	37/124	29.84	0.002
	Mixed (Baladi)	111/236	47.03	
Age	Foal (< 1 year)	9/23	39.13	0.005
	Young (1–4 years)	75/144	52.08	
	Adult (4–15 years)	58/170	34.12	
	Geriatric (≥15 years)	6/23	26.09	
Sex	Male	78/176	44.32	0.226
	Female	70/184	38.04	
Location	Cairo	30/100	30.00	0.008
	KFS	118/260	45.38	

KFS, Kafr El Sheikh.

Baladi: term used in Egypt for local mixed-breed animals.

**Table 3:** Results of the generalized estimating equations model identified potential risk factors associated with *T. gondii* exposure in equines.

**Variable**	**Category**	***P* value**	**OR^a^**	**95%CI**
Species	Donkey	< 0.001	2.99	2.35–3.81
	Horse	a	a	a

^a^Reference category.

## 4 Discussion

*Toxoplasma gondii* remains a highly prevalent zoonotic pathogen worldwide, affecting a broad spectrum of intermediate hosts, including equines. This far-reaching impact emphasizes the global significance of *T. gondii* infection (15, 19, 34), as equine toxoplasmosis is increasingly recognized as a major potential source of human infection (34). Unfortunately, there is a paucity of current data on the epidemiology of *T. gondii* infection in equids in Egypt, especially among donkeys. Most existing research is outdated and based on small sample sizes (15, 18, 19). This present study is considered one of the largest investigations into the seroprevalence of *T. gondii* in equids in Egypt, with a particular focus on donkeys.

The individual seroprevalence noted in horses in the current study (28.03%) is consistent with a previous study conducted in Cairo which reported a similar seroprevalence rate of 25% (25/100) (23). However, the present results are lower than those reported in various previous investigations in Cairo Governorate which showed seroprevalence values of 48.1% (202/420) (22) and 50.8% (122/240) (24). Another previous investigation reported an even higher seroprevalence rate in this species (72.2%; 39/54) in Dakahlia Governorate, Northern Egypt (15). In contrast, previous investigations reported a lower seroprevalences of 20.9% (23/110), 12.0% (12/100), 14.0% (14/100) and 17.3% (19/110) in horse populations in Giza, Qalyubia, Gharbia, and Kafr El Sheikh Governorates, respectively (25).

In the present study, the individual seroprevalence reported in donkeys was 51.23%, which is within the range previously recorded in this species in Egypt (Table 1). A nearly similar seroprevalence rate against *T. gondii* was observed in donkeys in Cairo Governorate (45%; 45/100) (21). However, our findings were lower than those reported in Menoufiya Governorate, Northern Egypt, where the seroprevalence was 65.3% (79/121) (18). Another study conducted in Dakahlia Governorate, Northern Egypt reported a higher seroprevalence rate of *T. gondii*, at 68.4% (54/79), among donkey populations in that area (15). In contrast, the current study found higher seroprevalence rates compared to those reported in Giza, Menoufiya, and Matrouh Governorates, Northern Egypt, using ELISA, where the seroprevalence rates were 37.9% (22/58), 25.6% (11/43), and 20.0% (9/45), respectively (19). The variation in *T. gondii* seroprevalence rates between the current study and previous research on equines could likely be attributed to several factors, primarily differences in farming management and sanitation practices, the specific serological tests used and the cutoff titers used for interpretation, timing of sampling, sample size, and the density of infected definitive hosts and their interaction with the animals (10, 15, 25, 35, 36). Likewise, climate-related factors such as geographic distribution, population density, and the abundance of cats (more prevalent in rural areas compared to urban governorates) would play a crucial role in the maintenance, spread, survival, and transmission of *T. gondii*, which contribute to the parasite's dynamics (2, 10, 37).

As shown in the present work, the risk of *T. gondii* seropositivity was 2.9 times greater in donkeys than in horses, indicating that donkeys are more susceptible to *T. gondii* exposure. These findings are consistent with previously reported data (38, 39). The variations in *T. gondii* seroprevalence rates between donkeys and horse may be attributed to differences in feeding, management, and sanitation practices including regular stall cleaning, disinfection, pasture rotation, proper manure disposal, and clean water supply (34). Donkeys in Egypt, often kept in extensive management systems under improper management and sanitation practices, and used more frequently for work, are more exposed to the parasite through ingestion of oocysts contaminating their environment, compared to horses (38). A previous investigation Munhoz et al. (39) concluded that donkeys could maintain detectable *T. gondii* antibody titers for a longer duration than horses. Additionally, the animal species susceptibility can also influence this difference (38, 40–43).

The current study has several limitations that should be noted. Firstly, the sample size from Cairo Governorate was small, and no donkey samples were collected in that area, which may limit the results' applicability to the overall equine population in this region. Cairo is a densely populated area with relatively few donkeys, which further constrains the extrapolation of our findings. Additionally, the study faced some challenges due to the small number of foals and geriatric equids, resulting in sample sizes variations between age groups. Some epidemiological factors, including breeding systems, sanitation conditions, and the presence of cats and rodents, were not assessed in this study. To gain a more comprehensive understanding, future large-scale serological and molecular studies should integrate these variables.

## 5 Conclusions

The present study constitutes one of the most extensive serosurveys of *T. gondii* in equines conducted in Egypt. The high level of exposure detected in these species raises animal and public health concerns. Notably, the study identified higher seroprevalence rates in donkeys compared to horses and represents the largest survey of donkeys ever conducted in Egypt so far. The study also offers new insights into the influence of various risk factors associated with the rate of exposure to the parasite. Given its significance for animal and public health, our seroepidemiological findings provide valuable information for Egyptian authorities to enhance control and prevention strategies for toxoplasmosis in equids. The study also underscores the importance of implementing strict measures to prevent potential transmission of the parasite to humans through the consumption of raw or undercooked meat and milk from the investigated species. Further large-scale serosurveys and molecular studies are warranted to genotype circulating *T. gondii* strains in equids and clarify their role in transmission to humans and animals in Egypt.

## Data Availability

The original contributions presented in the study are included in the article/supplementary material, further inquiries can be directed to the corresponding authors.
